# Prognostic associations of anthropometric indices with major health outcomes: A nationwide cohort study of 1.1 million adults

**DOI:** 10.1371/journal.pone.0359095

**Published:** 2026-09-24

**Authors:** Se-Jun Jeon, Sun-Hwa Kim, Si-Hyuck Kang, Tae-Jin Youn, In-Ho Chae

**Affiliations:** 1 Cardiovascular Center, Seoul National University Bundang Hospital, Seongnam-si, Korea; 2 Department of Internal Medicine, Seoul National University College of Medicine, Seoul, Korea; The Chinese University of Hong Kong, HONG KONG

## Abstract

**Background and Aims:**

The global escalation of obesity necessitates precise tools for assessing diverse health risks. This study aimed to compare the prognostic associations of body mass index (BMI), waist circumference (WC), and the body roundness index (BRI) across multiple cardiovascular and mortality outcomes in a large cohort of Korean adults using a nationwide database.

**Methods:**

This retrospective cohort study analyzed 1,199,633 Korean adults (aged ≥55 years) from the National Health Insurance Service database. Prognostic association for atherosclerotic cardiovascular disease (ASCVD), all-cause mortality, and atrial fibrillation (AF) over a 10-year follow-up was assessed using receiver operating characteristic (ROC) analysis and multivariable Cox proportional hazards models.

**Results:**

No single index consistently outperformed the others; rather, association was outcome-specific. BRI demonstrated the highest discrimination for ASCVD (AUC: 0.553) compared to WC (0.540) and BMI (0.512; P < 0.05 for all). Conversely, BMI showed the highest AUC for all-cause mortality (0.595), largely due to a significant inverse relationship in the underweight category. For incident AF, WC showed the highest AUC (0.567), followed by BRI (0.550) and BMI (0.527). Restricted cubic spline analyses revealed non-linear (U-shaped or J-shaped) relationships for most outcomes.

**Conclusions:**

These findings indicate that each anthropometric index captures distinct biological aspects of adiposity, highlighting the need to consider the specific clinical outcome when interpreting obesity-related risk.

## Introduction

The global prevalence of obesity continues to rise, and its association with a wide range of adverse health outcomes, including atherosclerotic cardiovascular disease (ASCVD), cancer, all-cause mortality, and atrial fibrillation (AF), is well established [1,2]. Traditionally, body mass index (BMI) has been the most widely used anthropometric index to define obesity, and several epidemiologic studies have demonstrated strong associations between BMI and both all-cause and disease-specific mortality, including cardiovascular and metabolic disorders [3–5].

However, BMI has been criticized for its limited ability to reflect body fat distribution, particularly visceral adiposity, which has emerged as a key determinant of cardiometabolic risk [6–9]. As central obesity gained recognition for its role in cardiovascular pathophysiology, several alternative indices incorporating waist circumference (WC) and body shape parameters have been proposed. The body roundness index (BRI), introduced in 2013, integrates height and WC to more accurately estimate body shape and visceral fat proportion. Recent studies have associated higher BRI values with increased risks of CVD, type 2 diabetes, metabolic syndrome, and chronic kidney disease [10–14]. A large cohort study of U.S. adults also reported an association between the BRI and all-cause mortality, highlighting its potential utility as a comprehensive marker of metabolic risk [15].

An important clinical question arising from these findings is whether any single anthropometric index is consistently more closely associated with diverse health outcomes, including ASCVD, cancer, and mortality. Most previous studies have focused on a single outcome or metabolic endpoint, and direct comparisons of BMI, WC, and BRI across multiple hard outcomes remain limited. Consequently, it remains unclear whether the prognostic associations of these indices differ according to specific outcomes, such as ASCVD, all-cause mortality, and disease-specific mortality. Therefore, the present study aimed to compare the prognostic associations of BMI, WC, and BRI across multiple cardiovascular and mortality endpoints in a large cohort of Korean adults using a nationwide database.

## Materials and methods

### Study design and population

This retrospective cohort analysis used data from the Korean National Health Insurance Service health screening cohort. Specifically, the data were captured from the national health screening program, a routine healthcare service provided by the government to all Korean citizens aged 40 and older. All participating clinics and hospitals are legally mandated to follow standardized protocols in accordance with the National Health Screening Act. Anthropometric measurements, including height, weight, and waist circumference, and laboratory blood tests are performed under strict quality control guidelines to ensure accuracy and standardization across all evaluation sites. These systematically standardized health examination records are then linked to longitudinal follow-up data. Data were accessed for research purposes between 30/01/2026 and 30/03/2026. Adults aged ≥55 years who underwent health screening between 2009 and 2012 were eligible for inclusion. From an initial sample of approximately 1.5 million individuals, we excluded participants with (1) a prior history of myocardial infarction (MI), stroke, heart failure or AF at baseline; (2) extreme anthropometric values (WC ≤ 40 cm or ≥130 cm, BMI ≥ 40 kg/m2, or height <130 cm); (3) missing data for key variables; or (4) invalid or unverified mortality records. Participants were followed for up to 10 years to ascertain outcomes.

Although cardiometabolic risk accumulates over decades, the age cut-off of ≥55 years was carefully selected based on national epidemiological data. According to the 2024 Cause of Death Statistics by Statistics Korea, the mortality rate from cardiovascular diseases is relatively low among individuals in their 40s, which limits the statistical power for evaluating long-term hard clinical endpoints. However, cardiovascular mortality and disease incidence increased exponentially starting in the mid-50s [16]. Therefore, we restricted our cohort to adults aged ≥55 years to ensure an adequate event rate and to focus on a clinically meaningful high-risk population where accumulated risks translate into actual cardiovascular events.

### Ethics approval and consent to participate

The study protocol was approved by the Institutional Review Board of Seoul National University Bundang Hospital (IRB No. X-2412-944-901) and was conducted in accordance with the principles of the Declaration of Helsinki. The requirement for informed consent was waived by the Institutional Review Board because this study used anonymized data from the Korean National Health Insurance Service database.

### Anthropometric indices

Height and weight were measured during standardized health examinations. The anthropometric indices evaluated in this study were BMI, WC, and BRI. BMI was calculated as weight (kg) divided by height squared (m^2^). WC was measured at the midpoint between the lower rib margin and the iliac crest. BRI was calculated using height (m) and WC (cm) according to a previously validated formula [10]:


$$\begin{array}{c} BRI=364.2-365.5\times \sqrt{1-{\left(\frac{WC}{2\pi }\right)}^{2}/{\left(0.5\times \text{height}\right)}^{2}} \end{array}$$

### Study outcomes

The primary study outcomes included ASCVD, all-cause mortality, and AF. ASCVD was defined as a composite outcome of cardiac death, MI, and ischemic stroke and was identified using hospitalisation records and International Classification of Diseases, 10th Revision (ICD-10) diagnostic codes. All-cause mortality was ascertained by linkage of individual identification codes to national death certificate records, including cause-of-death information. Incident AF was identified using hospitalisation records and ICD-10 diagnostic codes. The validity of using ICD-10 codes (I48) to identify AF in the Korean NHIS database has been previously validated with high accuracy [17]. Secondary outcomes included the individual components of ASCVD ─ cardiac death, MI, and ischemic stroke, as well as cancer-related mortality.

### Statistical analysis

Baseline risk factors included demographic characteristics (age and sex), lifestyle behaviours (smoking status), and comorbidities (hypertension, diabetes, and dyslipidaemia), ascertained through questionnaires, clinical measurements, and laboratory data. Baseline characteristics of the study population are presented as means ± standard deviations for continuous variables and percentages for categorical variables. The prognostic association of the anthropometric indices, BMI, WC, and BRI, was evaluated using receiver operating characteristic (ROC) curve analysis, with calculation of the area under the curve (AUC) for each study outcome. Although standard ROC analysis assumes a monotonic relationship, the AUC was utilized as the baseline metric because it serves as the most widely established standardized measure, enabling direct comparison with previous literature evaluating obesity indices [11–14]. To comprehensively address the limitations of ROC analysis in the presence of non-linear associations, we employed a complementary analytical approach. Specifically, restricted cubic spline (RCS) curves were constructed to examine potential non-linear associations, with knots placed at standard percentiles of the BRI distribution. Furthermore, Cox proportional hazards models were used to estimate hazard ratios (HRs) and 95% confidence intervals for incident outcomes across quintiles of each anthropometric index, after adjustment for age, sex, diabetes, hypertension, dyslipidemia, smoking status, and the Charlson comorbidity index. The third quintile (Q3) was selected as the reference category for all anthropometric indices because our restricted cubic spline analyses revealed prominent non-linear (U-shaped or J-shaped) associations, with the nadir of clinical risk consistently falling within the middle range (Q3). This approach allows for a clear and symmetrical comparison of increased risks at both the lower and higher extremes. All statistical analyses were performed using R software version 4.0.5, and statistical significance was set at P < 0.05.

## Results

The final study population comprised 1,199,633 adults aged ≥55 years who participated in routine health checkups (S1 Fig in S1 File). The mean age was 63.5 ± 7.3 years, and 47.2% of participants were male (Table 1). Mean systolic and diastolic blood pressures were 127.4 ± 15.8 and 78.0 ± 10.1 mmHg, respectively. Regarding comorbidities, 49.5% of participants had hypertension, 13.2% had diabetes mellitus, 48.8% had dyslipidaemia, and 16.2% were current smokers. Laboratory measurements showed mean values of 201.5 ± 42.7 mg/dL for total cholesterol, 120.3 ± 37.7 mg/dL for low-density lipoprotein cholesterol, 54.9 ± 31.8 mg/dL for high-density lipoprotein cholesterol, 141.7 ± 101.3 mg/dL for triglycerides, and 0.98 ± 1.04 mg/dL for serum creatinine. The mean values of the primary anthropometric indices were 24.04 ± 3.02 kg/m^2^, 82.4 ± 8.3 cm, and 3.75 ± 1.04 for BMI, WC, and BRI, respectively.

**Table 1:** Baseline characteristics of the study population.

Characteristics	Values
Age, years (n)	63.49 ± 7.30
Male sex, n (%)	566,526 (47.2%)
Anthropometric measurements, (n)	
Body mass index, kg/m^2 †^	24.04 ± 3.02
Systolic blood pressure, mmHg	127.37 ± 15.84
Diastolic blood pressure, mmHg	77.98 ± 10.05
Comorbidities, n (%)	
Hypertension	593,468 (49.5%)
Diabetes mellitus	157,907 (13.2%)
Dyslipidemia	585,396 (48.8%)
Current smoker	194,907 (16.2%)
Laboratory findings, mg/dL (n)	
Total cholesterol	201.53 ± 42.72
HDL cholesterol	54.92 ± 31.79
LDL cholesterol	120.27 ± 37.68
Triglyceride	141.66 ± 101.29
Serum creatinine	0.98 ± 1.04

During the 10-year follow-up period, ASCVD occurred in 9.6% of the study population, including 1.9% of cardiac deaths, 3.3% of MI, and 4.2% of ischemic stroke. The cumulative incidences of all-cause and cancer-related mortalities were 10.9% and 4.2%, respectively. Additionally, 4.7% of the study population developed AF (S1 Table in S1 File).

**Fig 1:**
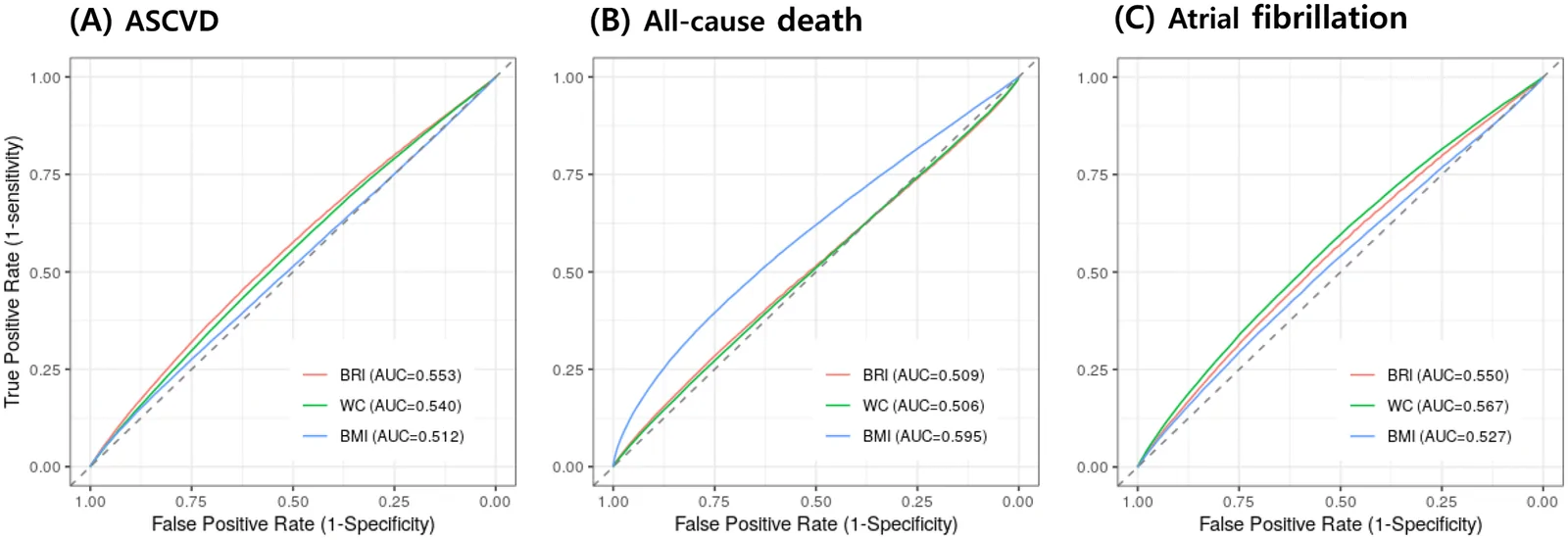
Receiver operating characteristic curves comparing the prognostic performance of BMI, WC and BRI for major clinical outcomes.

The prognostic performance of the three anthropometric indices was compared using ROC curve analysis (Fig 1). For the primary outcome of ASCVD, the AUC was highest for the BRI at 0.553, followed by WC at 0.540 and BMI at 0.512 (P < 0.05 for all comparisons). In contrast, BMI demonstrated a significantly higher AUC for all-cause mortality than BRI and WC (0.595 vs. 0.509 and 0.506, respectively; P < 0.05 for all). For AF, WC showed the highest AUC, followed by BRI and BMI (0.567, 0.550, and 0.527, respectively; P < 0.05 for all). ROC analyses for individual ASCVD components and cancer-related mortality are presented in S2 Fig in S1 File. Detailed ROC analyses for individual outcomes are presented in S2 Table in S1 File.

**Fig 2:**
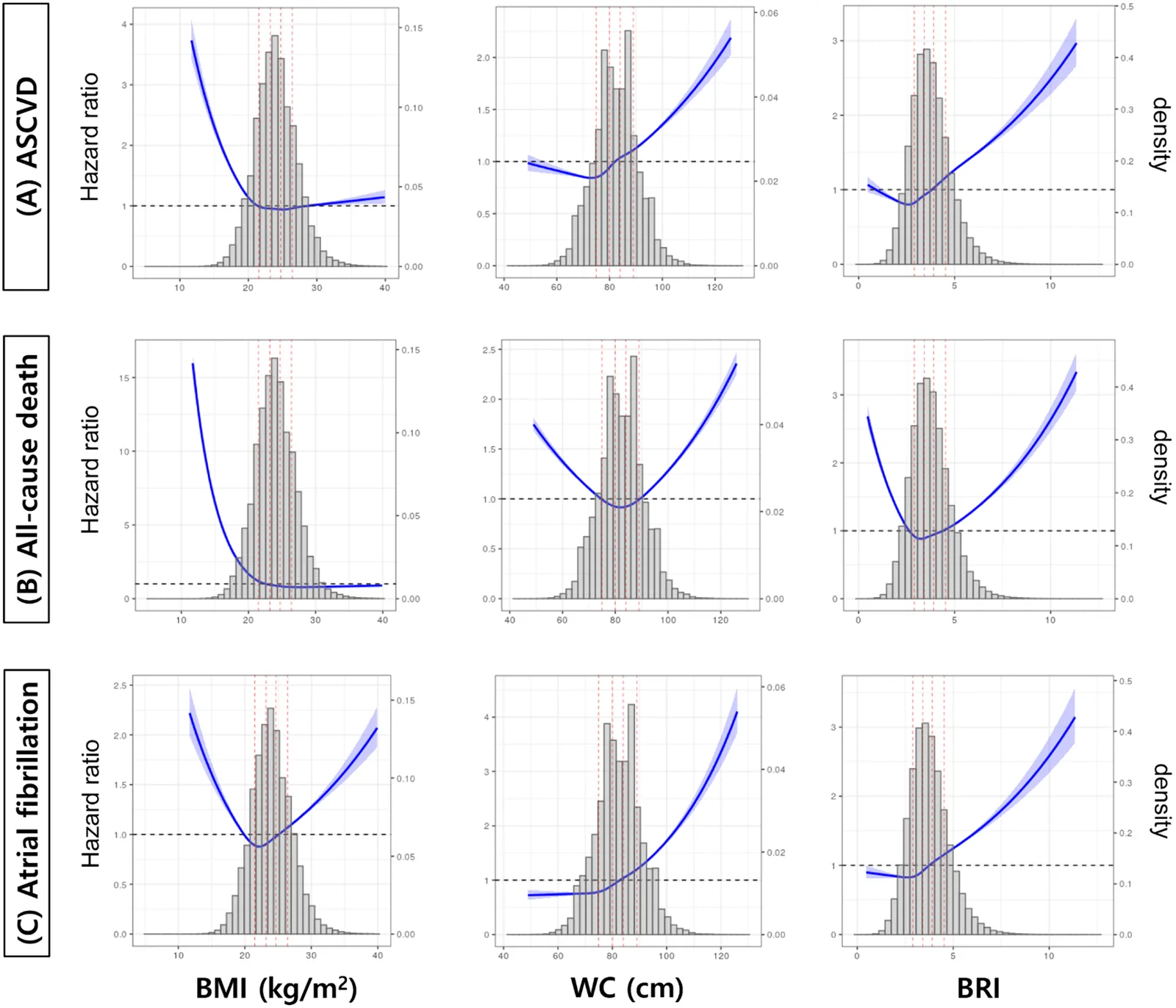
Restricted cubic spline analyses showing associations between anthropometric indices and clinical outcomes. Dashed horizontal lines indicate a hazard ratio of 1.0. Vertical dashed red lines represent quintile cut-off points.

Fig 2 presents RCS curves illustrating the relationships between each anthropometric index and the study outcomes. BMI demonstrated a strong inverse association with ASCVD in the underweight range, whereas the positive association in the obese range was less prominent. WC and BRI exhibited J-shaped relationships with ASCVD, with nadirs at 72 cm and 2.6, respectively, and relatively short negative tails in the underweight range. For all-cause mortality, BMI showed a strong inverse association in the underweight range, whereas WC and BRI demonstrated U-shaped relationships with nadirs at 80 cm and 2.6, respectively. The association between BMI and AF was U-shaped, with a nadir at 22–23 kg/m^2^. In contrast, WC and BRI showed a J-shaped relationship with AF, with relatively flat tails in the underweight range. Corresponding RCS analyses for individual ASCVD components and cancer-related mortality are shown in S3 Fig in S1 File.

HRs adjusted for baseline risk factors across quintiles are presented in Table 2. After multivariable adjustment, the association between BMI and ASCVD was largely attenuated, with only a modest increase in risk observed in the highest quintile. In contrast, the BRI demonstrated a clear and consistent positive association with ASCVD. The associations of all-cause mortality and AF were generally consistent with those observed in the unadjusted analyses. Lower anthropometric index values were associated with a higher risk of all-cause mortality, whereas only the BRI showed an elevated risk in the highest quintile. After multivariable adjustment, all three anthropometric indices were positively associated with AF, with WC showing the strongest association.

**Table 2:** Hazard ratios (HRs) with 95% confidence intervals (CIs) derived from cox proportional hazard models for atherosclerotic cardiovascular disease, all-cause death and atrial fibrillation according to quintiles of BMI, WC and BRI.

		Q1	Q2	Q3	Q4	Q5
Atherosclerotic cardiovascular disease	BMI	1.07 (1.05–1.09)	1.00 (0.98–1.02)	Ref	0.99 (0.98–1.01)	1.04 (1.02–1.06)
	WC	0.97 (0.95–0.99)	0.97 (0.95–0.99)	Ref	1.02 (1.00–1.04)	1.05 (1.03–1.07)
	BRI	0.94 (0.92–0.96)	0.97 (0.95–0.98)	Ref	1.04 (1.03–1.06)	1.11 (1.09–1.13)
All-cause death	BMI	1.57 (1.54–1.59)	1.12 (1.10–1.14)	Ref	0.95 (0.93–0.97)	0.98 (0.96–1.00)
	WC	1.32 (1.29–1.34)	1.08 (1.07–1.10)	Ref	0.96 (0.94–0.98)	0.97 (0.95–0.99)
	BRI	1.28 (1.26–1.30)	1.06 (1.04–1.08)	Ref	0.98 (0.97–1.00)	1.04 (1.02–1.06)
Atrial fibrillation	BMI	0.97 (0.95-1.00)	0.96 (0.93-0.98)	Ref	1.07 (1.04-1.10)	1.25 (1.22-1.28)
	WC	0.89 (0.87–0.92)	0.93 (0.90–0.95)	Ref	1.04 (1.02–1.07)	1.24 (1.21–1.27)
	BRI	0.94 (0.92–0.97)	0.96 (0.93–0.99)	Ref	1.03 (1.01–1.06)	1.17 (1.14–1.20)

*Q3 was used as the reference category. **Models were adjusted for age, sex, diabetes mellitus, hypertension, dyslipidemia, smoking status, and Charlson Comorbidity Index (CCI).

To address the possibility of reverse causality, specifically, the concern that the high mortality observed in the lower ranges of anthropometric indices might reflect pre-existing or pre-clinical illnesses leading to unintentional weight loss, we conducted a landmark analysis. We excluded participants who died or experienced major clinical events within the first 1 and 2 years of follow-up. As shown in S3 and S4 Tables, and S4 and S5 Figs in S1 File, the associations (AUCs) and the non-linear risk patterns (hazard ratios) of the anthropometric indices for clinical outcomes remained robust and highly consistent with our primary findings. This persistence of risk patterns, even after excluding early events, suggests that the observed increased risks at the lower extremes of these indices are not merely driven by early reverse causation.

## Discussion

This study analyzed a large nationwide cohort of Korean adults to compare the prognostic value of the three most commonly used anthropometric indices for obesity, BMI, WC, and BRI, across major health outcomes. A key finding was that the relative performance of these indices varied significantly depending on the clinical endpoint of interest. The BRI demonstrated the best discrimination for ASCVD, showing a consistent positive association. Although BMI showed the highest AUC for mortality, its inverse association in the underweight group was stronger than its positive association in the overweight and obese categories. For AF, WC showed the highest discriminatory ability, followed by BRI and BMI. These findings indicate that no single anthropometric index universally outperforms the others in terms of major health outcomes.

One notable finding of this study was that the prognostic associations of each anthropometric index on clinical outcomes was modest, with AUC values not exceeding 0.60. Although obesity is closely associated with cardiometabolic disorders, such as hypertension, diabetes, dyslipidaemia, and insulin resistance [18–20], its impact on hard clinical endpoints, including ASCVD and mortality, appears to be limited [21,22]. Statistical modeling has established that cardiometabolic risk factors, such as hypertension, diabetes, and dyslipidaemia, exhibit superior prognostic value for clinical outcomes than anthropometric indices alone [23,24]. Consistent with these findings, we observed that the associations between anthropometric indices and outcomes were largely attenuated after multivariable adjustment.

The non-linear associations between anthropometric indices and clinical outcomes likely contributed to the modest AUC values observed in the present study. We identified varying degrees of J- or U-shaped relationships across major health outcomes. Previous studies have demonstrated that ROC curves tend toward a null discrimination when a continuous variable exhibits a bimodal or U-shaped association with an outcome [25]. Consequently, for outcomes, such as ASCVD and mortality, the concurrent increase in risk at both extremes of the anthropometric spectrum attenuates the ability of any single index to effectively discriminate risk.

The differential associations observed among the three anthropometric indices likely reflect the distinct physiological processes captured by each measure. ASCVD is strongly associated with visceral adiposity-related inflammation, insulin resistance, and metabolic dysfunction [26]. These mechanisms are better captured by the BRI, which incorporates WC relative to height and more closely reflects visceral adiposity. Accordingly, the BRI showed a consistently positive association with ASCVD in both crude and multivariable analyses. In contrast, mortality outcomes were more closely associated with BMI, which reflects overall body mass and is influenced not only by excess fat but also by age-related frailty and involuntary weight loss. These factors likely contributed to the observed U-shaped relationship between BMI and mortality, consistent with findings from previous studies examining body size and mortality risk [27–29]. In contrast, AF is closely associated with abdominal obesity, atrial stretch, and volume overload [30,31]. Because WC directly reflects central fat accumulation, it demonstrated the highest association with AF in our analyses. Collectively, these findings suggest that each anthropometric index captures a distinct biological dimension of adiposity, resulting in outcome-specific differences in prognostic associations.

This study has several strengths. It was conducted in a large nationwide cohort with standardized baseline measurements and extended follow-up, enabling a reliable estimation of multiple cardiovascular outcomes. Few previous studies have directly compared BRI, WC, and BMI within the same population across diverse clinical endpoints, including ASCVD, all-cause mortality, disease-specific mortality, and AF.

## Limitation

This study has several limitations. First, anthropometric indices were assessed only at baseline, and potential changes in body composition over time were not captured. Recent evidence indicates that pharmacological interventions, such as glucagon-like peptide-1 receptor agonists, can improve cardiovascular outcomes in overweight and obese individuals, highlighting the importance of dynamic risk assessment [32,33]. Second, the study population comprised Korean adults aged ≥55 years, which may limit the generalisability of the findings to younger populations or other ethnic groups. Third, direct measures of adiposity, such as computed tomography- or magnetic resonance imaging-based assessments of visceral fat, were not available and would have allowed for more precise characterisation of fat distribution. Fourth, despite comprehensive adjustment for potential covariates including age, sex, smoking status, and Charlson Comorbidity Index (CCI), the possibility of residual confounding from unmeasured lifestyle factors cannot be entirely ruled out. Specifically, detailed data on physical activity, dietary habits, and adherence to key medications such as statins or antihypertensive drugs, were not fully adjusted. Additionally, socioeconomic status, which is often represented by insurance premiums in the National Health Insurance Service database, could not be reliably integrated due to substantial missing values and methodological difficulties in standardized categorization.

Fifth, the anthropometric indices were evaluated solely based on a single measurement at baseline. Over a 10-year follow-up period in this aging cohort, individuals are expected to experience substantial dynamic changes in body composition such as weight fluctuations, redistribution of body fat, or progressive muscle loss. Because our study could not capture these longitudinal variations, it may have introduced some degree of exposure misclassification over time. Finally, all AUC values were <0.6, indicating that no single anthropometric index provided strong discriminatory power for cardiovascular or mortality outcomes. This likely reflects the biological reality that obesity is a distal risk factor, with its effects on ASCVD and mortality mediated through complex metabolic and clinical pathways. Additionally, standard ROC analysis assumes a monotonic relationship between the variable and the outcome; therefore, the use of AUC to compare prognostic association may be inherently limited and potentially problematic when the underlying risk increases at both extremes, such as underweight and severe obesity.

## Conclusion

In this large nationwide cohort, the associations of anthropometric indices varied according to clinical outcomes. The BRI demonstrated a closer association for ASCVD than WC and BMI, whereas BMI and WC showed the most prominent associations with all-cause mortality and AF, respectively. Although the absolute associations were modest and characterized by non-linear relationships, these findings underscore that no single anthropometric index is sufficient for a comprehensive risk assessment, reflecting the complex interplay between adiposity and major health outcomes.

## Supporting information

S1 File Supporting information. Contains S1 Fig (Flow diagram of the study population selection), S2 Fig (ROC analysis for individual components of ASCVD and cancer death), S3 Fig (Restricted cubic spline analyses for individual components), S4 Fig (RCS in 1-year landmark analysis), S5 Fig (RCS in 2-year landmark analysis), S1 Table (Event rates of ASCVD, death, and AF), S2 Table (ROC analysis of study variables and outcomes), S3 Table (Comparison of AUC in 1-year and 2-year landmark analyses), and S4 Table (Multivariable-adjusted hazard ratios in landmark analyses).
(DOCX)
