# Supplementary material for: Prognostic associations of anthropometric indices with major health outcomes: A nationwide cohort study of 1.1 million adults
Source: PLoS One. 2026 Sep 24;21(9):e0359095. doi: 10.1371/journal.pone.0359095 (PMC13606998; doi:10.1371/journal.pone.0359095)
Supplement: Supplementary file 1 [file pone.0359095.s001.docx]

**Supplementary Figure 1.** Flow diagram of the study population selection

**Supplementary Table 1.** Event rates of atherosclerotic cardiovascular disease (ASCVD), death, and atrial fibrillation (AF) among the study population (n=1,199,633) for 10 years

| Events | Number of events (%) |
| --- | --- |
| Atherosclerotic cardiovascular disease | 115,373 (9.6%) |
| Cardiac death | 22,824 (1.9%) |
| Myocardial infarction | 39,240 (3.3%) |
| Ischemic stroke | 49,802 (4.2%) |
| All-cause death | 130,815 (10.9%) |
| Cancer death | 49,802 (4.2%) |
| Atrial fibrillation | 55,800 (4.7%) |

**Supplementary Table 2.** Receiver operating characteristic analysis of study variables and outcomes

|  | BMI | WC | BRI |
| --- | --- | --- | --- |
| Atherosclerotic cardiovascular disease | 0.512 (0.510–0.514) | 0.540 (0.538–0.541) | 0.553 (0.551–0.554) |
| Cardiac death | 0.576 (0.572–0.580) | 0.510 (0.506–0.514) | 0.542 (0.538–0.546) |
| Myocardial infarction | 0.505 (0.502–0.508) | 0.545 (0.543–0.548) | 0.549 (0.547–0.552) |
| Ischemic stroke | 0.494 (0.492–0.496) | 0.542 (0.539–0.544) | 0.556 (0.554–0.558) |
| All-cause death | 0.595 (0.593–0.596) | 0.506 (0.504–0.508) | 0.509 (0.508–0.511) |
| Cancer death | 0.553 (0.550–0.556) | 0.529 (0.527–0.532) | 0.504 (0.501–0.507) |
| Atrial fibrillation | 0.527 (0.524–0.529) | 0.567 (0.565–0.570) | 0.550 (0.547–0.552) |

**Supplementary Figure 2.** Receiver operating characteristic analysis of anthropometric indices for individual components of atherosclerotic cardiovascular disease and cancer death

**Supplementary Figure 3.** Restricted cubic spline analyses showing associations between anthropometric indices and individual components of atherosclerotic cardiovascular disease and cancer death

**Supplementary Table 3.** Comparison of area under the receiver operating characteristic curves (AUC) of anthropometric indices for primary clinical outcomes occurring after 1 year and 2 years in landmark analyses.

|  |  | | BMI | WC | BRI |
| --- | --- | --- | --- | --- | --- |
| 1-Year Landmark | | Atherosclerotic cardiovascular disease | 0.512 (0.510-0.513) | 0.539 (0.537-0.541) | 0.552 (0.550-0.554) |
|  |  | Cardiac death | 0.575 (0.571-0.579) | 0.512 (0.508-0.516) | 0.544 (0.540-0.548) |
|  |  | Myocardial infarction | 0.506 (0.502-0.509) | 0.545 (0.542-0.548) | 0.549 (0.546-0.552) |
|  |  | Ischemic stroke | 0.494 (0.491-0.496) | 0.541 (0.539-0.543) | 0.555 (0.553-0.557) |
|  |  | All-cause death | 0.591 (0.589-0.592) | 0.509 (0.507-0.511) | 0.513 (0.511-0.515) |
|  |  | Cancer death | 0.547 (0.544-0.550) | 0.533 (0.530-0.536) | 0.508 (0.505-0.510) |
|  |  | Atrial fibrillation | 0.527 (0.524-0.529) | 0.568 (0.565-0.570) | 0.550 (0.547-0.553) |
| 2-Year Landmark | | Atherosclerotic cardiovascular disease | 0.489 (0.487-0.491) | 0.539 (0.537-0.541) | 0.552 (0.550-0.554) |
|  |  | Cardiac death | 0.573 (0.569-0.577) | 0.513 (0.509-0.517) | 0.546 (0.542-0.550) |
|  |  | Myocardial infarction | 0.505 (0.502-0.509) | 0.544 (0.541-0.547) | 0.549 (0.546-0.552) |
|  |  | Ischemic stroke | 0.494 (0.492-0.497) | 0.541 (0.538-0.543) | 0.554 (0.552-0.557) |
|  |  | All-cause death | 0.587 (0.585-0.589) | 0.511 (0.509-0.513) | 0.516 (0.514-0.517) |
|  |  | Cancer death | 0.543 (0.540-0.546) | 0.536 (0.533-0.538) | 0.510 (0.507-0.513) |
|  |  | Atrial fibrillation | 0.526 (0.524-0.529) | 0.567 (0.564-0.570) | 0.549 (0.547-0.552) |

**Supplementary Table 4.** Multivariable-adjusted hazard ratios (95% confidence intervals) for clinical outcomes according to quintiles of BMI, WC, and BRI occurring after 1 year and 2 years in landmark analyses.

|  |  |  | Q1 | Q2 | Q3 | Q4 | Q5 |
| --- | --- | --- | --- | --- | --- | --- | --- |
| 1-Year Landmark | Atherosclerotic cardiovascular disease | BMI | 1.07 (1.05-1.09) | 0.99 (0.97-1.01) | Ref | 0.99 (0.97-1.01) | 1.03 (1.01-1.05) |
|  |  | WC | 0.97 (0.95-0.99) | 0.97 (0.95-0.99) | Ref | 1.02 (1.00-1.04) | 1.05 (1.03-1.06) |
|  |  | BRI | 0.93 (0.91-0.95) | 0.97 (0.95-0.98) | Ref | 1.04 (1.02-1.06) | 1.11 (1.09-1.13) |
|  | All-cause death | BMI | 1.53 (1.51-1.56) | 1.12 (1.10-1.14) | Ref | 0.95 (0.93-0.97) | 0.98 (0.96-1.00) |
|  |  | WC | 1.29 (1.27-1.31) | 1.08 (1.06-1.10) | Ref | 0.96 (0.94-0.98) | 0.98 (0.96-1.00) |
|  |  | BRI | 1.26 (1.23-1.28) | 1.06 (1.04-1.08) | Ref | 0.99 (0.97-1.01) | 1.05 (1.03-1.07) |
|  | Atrial fibrillation | BMI | 0.97 (0.95-1.00) | 0.95 (0.93-0.98) | Ref | 1.07 (1.05-1.10) | 1.25 (1.22-1.29) |
|  |  | WC | 0.89 (0.86-0.91) | 0.92 (0.90-0.95) | Ref | 1.04 (1.01-1.07) | 1.24 (1.21-1.27) |
|  |  | BRI | 0.94 (0.92-0.97) | 0.96 (0.94-0.99) | Ref | 1.03 (1.01-1.06) | 1.18 (1.15-1.21) |
| 2-Year Landmark | Atherosclerotic cardiovascular disease | BMI | 1.07 (1.04-1.09) | 0.99 (0.97-1.01) | Ref | 0.99 (0.97-1.01) | 1.04 (1.02-1.06) |
|  |  | WC | 0.97 (0.95-0.99) | 0.96 (0.94-0.98) | Ref | 1.01 (0.99-1.03) | 1.04 (1.02-1.06) |
|  |  | BRI | 0.93 (0.91-0.95) | 0.96 (0.94-0.98) | Ref | 1.04 (1.02-1.06) | 1.11 (1.09-1.13) |
|  | All-cause death | BMI | 1.51 (1.49-1.54) | 1.11 (1.09-1.13) | Ref | 0.95 (0.93-0.97) | 0.99 (0.97-1.01) |
|  |  | WC | 1.28 (1.26-1.30) | 1.08 (1.06-1.10) | Ref | 0.97 (0.95-0.98) | 0.99 (0.97-1.00) |
|  |  | BRI | 1.24 (1.22-1.27) | 1.05 (1.03-1.07) | Ref | 0.95 (0.97-1.01) | 1.05 (1.04-1.07) |
|  | Atrial fibrillation | BMI | 0.98 (0.95-1.01) | 0.95 (0.93-0.98) | Ref | 1.08 (1.05-1.11) | 1.26 (1.22-1.29) |
|  |  | WC | 0.89 (0.86-0.92) | 0.93 (0.90-0.96) | Ref | 1.04 (1.01-1.07) | 1.24 (1.21-1.28) |
|  |  | BRI | 0.95 (0.92-0.98) | 0.97 (0.94-1.00) | Ref | 1.04 (1.01-1.07) | 1.18 (1.15-1.21) |

***Q3 was used as the reference category. **Models were adjusted for age, sex, diabetes mellitus, hypertension, dyslipidemia, smoking status, and Charlson Comorbidity Index (CCI) ***1-year landmark analysis excluded participants who died or experienced clinical events within the first year of follow-up. 2-year landmark analysis excluded those within the first two years of follow-up.**

**Supplementary Figure 4.** Restricted cubic spline analyses showing associations between anthropometric indices and primary clinical outcomes in 1-year landmark analysis.

**Supplementary Figure 5.** Restricted cubic spline analyses showing associations between anthropometric indices and primary clinical outcomes in 2-year landmark analysis.
